# Defect-Related Photoluminescence in Hydroxyapatite Nanoparticles Modulated by Carbonate Incorporation

**DOI:** 10.1021/acsnanoscienceau.5c00140

**Published:** 2025-12-18

**Authors:** Thales R. Machado, Lívia G. Pacífico, Marylyn S. Arai, Beatriz G. R. da Silva, Angélica M. M. Zapata, Raquel R. C. Vilela, Valtencir Zucolotto

**Affiliations:** GNANO—Nanomedicine and Nanotoxicology Group, São Carlos Institute of Physics, 117186 University of São Paulo, IFSC—USP, 13566-590 São Carlos, São Paulo, Brazil

**Keywords:** hydroxyapatite, luminescence, defects, carbonates, bioimaging, nanoparticles, nanomedicine

## Abstract

The investigation of defect-related photoluminescence in hydroxyapatite (HA) nanoparticles (NPs) is essential for understanding their electronic structure and charge carrier recombination dynamics. These insights are important to advancing HA-based materials in photocatalysis, optical devices, hard tissue spectroscopy, and cellular bioimaging. In this study, we provide new evidence on the structural and compositional factors that govern the intrinsic photoluminescence of HA NPs synthesized by chemical precipitation at room temperature, and subjected to thermal treatment at 400 and 450 °C. Carbonate contents ranging from 0.6 to 10.9 wt % were introduced into HA nanorods during synthesis through AB-type substitution, replacing both OH^–^ (A-type) and PO_4_^3–^ (B-type) groups. Increasing carbonate incorporation led to enhanced emissions under 405 nm excitation, with a primary band centered at 438 nm. Subsequent thermal treatment further amplified the emission intensity, with the strongest luminescence observed in samples containing higher carbonate content, which also exhibited a red-shift of the emission maximum to approximately 583 nm. These changes were mainly attributed to the progressive increase in carbonate concentration, particularly through B-type substitution, which promotes structural disorder, reduces crystallite size, and generates higher densities of vacancies defects, including V_Ca_, V_OH_, and V_O_ in PO_4_^3–^ groups, as well as to the elimination of structural water during heating. These results confirm that carbonate, a frequent impurity in almost all HA NPs obtained by wet methods without strict experimental conditions (e.g., inert atmosphere), plays a central role in modulating the density of defects and, consequently, the photoluminescence properties in both as-synthesized and thermally treated forms. We also demonstrate the use of citrate-functionalized carbonated HA NPs for cellular bioimaging with HDFn cells as a model, underscoring their potential for biomedical applications.

## Graphical abstract

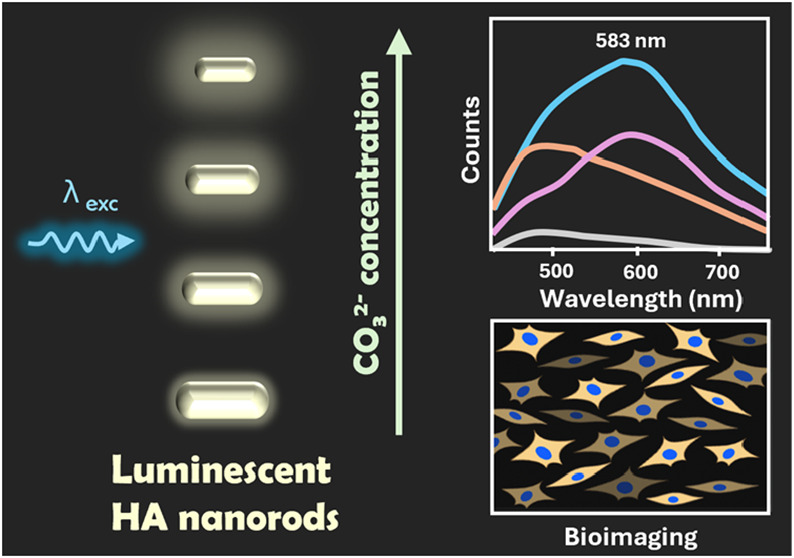

## 1 Introduction

Hydroxyapatite (HA, Ca_10_(PO_4_)_6_(OH)_2_) nanoparticles (NPs) are widely studied for biomedical applications such as bone repair,^1,2^ tissue engineering,^3^ and drug delivery,^4^ owing to their high biocompatibility, bioactivity, and chemical similarities to biogenic apatite NPs found in hard tissues.^5^ Beyond this context, HA NPs have garnered attention for their optical properties, particularly their defect-related photoluminescence.^6–11^ Understanding and modulating this property has proven important not only for the design of HA-fluorescent probes in bioimaging^12–14^ and apatite-based optical devices,^15,16^ but also for emerging applications of HA-based heterostructures in photocatalysis for environmental remediation and bacterial disinfection, where charge carrier recombination dynamics play a central role.^17–19^ Additionally, the photoluminescence of synthetic HA NPs has been explored to support the spectroscopic analysis of bones and fossils.^20,21^ These diverse applications underscore the growing importance of investigating the photoluminescent behavior of HA NPs and its correlation with structural and compositional features.

The origins of the intrinsic photoluminescence in HA NPs have been previously investigated.^22^ The broad emission band observed is mainly attributed to the recombination of electron–hole (e^–^–h^+^) pairs involving multiple defect-related energy states within the bandgap (*E*_g_ = 5.5–5.7 eV), arising from the intrinsically defective nature of HA.^23,24^ These states result from structural disorder in the bulk, surface, and interfaces, including bonding distortions that lead to perturbations in the electronic density of the [CaO_9_], [CaO_6_(OH)], and [PO_4_] coordination polyhedra, as well as the presence of ionic vacancies, such as calcium vacancies (V_Ca_), hydroxyl vacancies (V_OH_), and oxygen vacancies (V_O_) within phosphate sites, along with hydrogen interstitials.^23,24^ Theoretical studies further support the influence of these defects on the electronic structure of HA, particularly in the bandgap region.^25–27^

Among the structural imperfections in HA, carbonate ions (CO_3_^2–^) represent one of the most common substitutional defects, accounting for 2–8 wt % in biogenic apatite NPs from hard tissues.^28,29^ Almost any synthesis of HA under atmospheric conditions naturally leads to its incorporation, as the substitution of PO_4_^3–^ or OH^–^ groups by CO_3_^2–^ is thermodynamically favorable.^30,31^ Such substitutions require charge-compensating vacancies and lattice distortions, increasing structural disorder and reducing crystal size.^29^ Consequently, CO_3_^2–^ incorporation directly influences the crystallinity, solubility and biological behavior of HA NPs,^32^ which can be tuned by using a CO_3_^2–^ precursor for applications such as pH-responsive drug delivery systems,^33^ and bone repair/substitution.^34–36^ Furthermore, carbonated HA undergoes temperature-dependent transformations upon heating, including CO_3_^2–^ reorganization and release,^37–39^ which potentially alter its *in vivo* behavior and electronic properties. In this way, understanding the impact of CO_3_^2–^ on the photoluminescence of as-synthesized and heat-treated HA is essential to elucidate the defect chemistry and emission mechanisms of this biomaterial, as well as to design biodegradable photoluminescent HA NPs with compositions closer to biogenic apatite. These features could enhance their potential for biomedical and technological applications and further support biogenic apatite characterization.

It has been reported that CO_3_^2–^ contributes to the enhanced photoluminescence observed in HA NPs after thermal treatment at 350–450 °C, compared to the as-synthesized samples.^40,41^ Subsequent investigations concluded that changes in synthesis conditions at room temperature (RT) in aqueous solution modulate the CO_3_^2–^ content and associated defects in HA NPs and, consequently, their photoluminescence properties after heat treatment.^42–44^ Although these later studies have contributed significantly to advances in the field, they primarily focused on photoluminescence changes following thermal treatment, while the as-synthesized samples were either not reported, exhibited negligible emission, or were not thoroughly characterized. Investigating this aspect could clarify the role of CO_3_^2–^ and the associated stabilizing defects in the HA lattice, without interference from thermally induced structural or compositional changes. Moreover, the previous studies did not quantify carbonate content and typically introduced it indirectly, at varying levels, by altering parameters such as the chemical precipitation pathway or pH.^42–44^ These additional variables affect the structure, morphology, and composition of HA,^45,46^ thereby potentially influencing the optical properties of both the as-synthesized and heat-treated NPs, and confounding the specific effects of CO_3_^2–^ incorporation.

In this study, we synthesized HA NPs by a commonly employed aqueous precipitation under atmospheric conditions, which often results in CO_3_^2–^ incorporation.^38,47^ We then compared these particles with HA NPs prepared with varying carbonate contents by directly adding a CO_3_^2–^ precursor over a broad [CO_3_^2–^]/[PO_4_^3–^] molar ratio range (0.0625–4), while keeping all other synthesis parameters constant. We demonstrate that this strategy enables a precise evaluation of the correlation between carbonate incorporation, defect chemistry, and intrinsic photoluminescence of HA NPs, both before and after heat treatment. Building on our previous methodology applied to carbonated amorphous calcium phosphate (ACP) NPs,^48^ we now extend this approach to carbonated HA NPs. This study also demonstrates the potential of citrate functionalized HA NPs with optimized photoluminescence, achieved through CO_3_^2–^ incorporation at levels comparable to biogenic apatite (7.9 wt %) and heat treatment at 450 °C, for bioimaging applications.

## 2 Materials and Methods

### 2.1 Synthesis of Carbonated HA NPs

The HA sample prepared without a CO_3_^2–^ precursor was synthesized by chemical precipitation in aqueous solution at RT using a procedure adapted from our previous protocol.^41^ Initially, 10 mmol of calcium nitrate tetrahydrate (Ca­(NO_3_)_2_·4H_2_O, 99%, Sigma-Aldrich) was dissolved in 50 mL of Milli-Q H_2_O (Solution 1), and 6 mmol of diammonium hydrogen phosphate ((NH_4_)_2_HPO_4_, 98+%, Sigma-Aldrich) in 100 mL of Milli-Q H_2_O (Solution 2). The pH of both solutions was adjusted to 10 using concentrated ammonium hydroxide (NH_4_OH, 28%, LabSynth). Solution 2 was then rapidly poured into Solution 1 under stirring, resulting in a white suspension that was stirred continuously for 24 h. To remove potential unreacted Ca^2+^ and phosphate ions, together with NH_4_^+^ and NO_3_^–^, the suspension was divided into four 50 mL Falcon tubes and centrifuged at 8000 rpm for 3 min. The resulting pellets were purified by resuspending each in 40 mL of ultrapure water, a process repeated six times (960 mL in total), followed by a final step with absolute ethanol (160 mL in total). The resulting NPs were dried in an oven at 80 °C for approximately 24 h, ground using an agate mortar and pestle without sieving, and stored at 4 °C. This sample was referred to as HAS1.

For the preparation of HA NPs containing varied concentrations of CO_3_^2–^, the same procedure was employed, except that ammonium carbonate ((NH_4_)_2_CO_3_, 99%, Sigma-Aldrich) was added to Solution 2 at [CO_3_^2–^]/[PO_4_^3–^] molar ratios of 0.0625, 0.125, 0.25, 0.5, 1, 2, and 4, while maintaining a fixed Ca/P molar ratio of 1.667. After synthesis, free carbonate ions, along with Ca^2+^, PO_4_^3–^, NH_4_^+^ and NO_3_^–^, were removed by consecutive centrifugation and pellet resuspension, following the same procedure used for the HAS1 sample. The obtained NPs were subsequently subjected to drying, grinding, and storage. The resulting samples were labeled as HAS2 to HAS8, as summarized in Table 1.

**Table 1:** Summary of the Carbonated HA NPs Prepared by Chemical Precipitation at RT with Varying [CO_3_^2–^]/[PO_4_^3–^] Molar Ratio, along with the Corresponding Crystallite Sizes Values Estimated from XRD Data

sample	[CO_3_^2–^]/[PO_4_^3–^] molar ratio	*D*_002_ (nm)	*D*_300_ (nm)	*D*_002_/*D*_300_
HAS1	-	41	19	2.2
HAS2	0.0625	39	18	2.2
HAS3	0.125	44	21	2.1
HAS4	0.25	31	19	1.6
HAS5	0.5	34	17	2.0
HAS6	1	25	15	1.7
HAS7	2	21	12	1.7
HAS8	4	21	14	1.5

### 2.2 Heat Treatment

The as-synthesized samples were thermally treated in a 3P–S muffle furnace (EDG Equipment, Brazil) under ambient atmosphere at either 400 or 450 °C, using a heating rate of 10 °C min^–1^. The samples were maintained at the selected temperature for 4 h, followed by natural cooling to room temperature. The resulting materials were labeled HAS1–400 to HAS8–400 or HAS1–450 to HAS8–450, according to the treatment temperature.

### 2.3 Characterizations

X-ray diffraction (XRD) analysis was employed to identify the crystalline phases present in the as-synthesized carbonated HA samples. Measurements were conducted using a DMax2500PC diffractometer (Rigaku) with Cu–Kα radiation (λ = 0.154184 nm), operating at a scanning rate of 0.02°/s over a 2θ range of 20° to 80°. The crystallite sizes (*D*) of as-synthesized NPs were estimated for the (002) and (300) reflection planes using the Scherrer equation^49^

1 $$D=\frac{0.89\cdot \lambda }{\beta \cdot \cos\theta }$$

where λ is the X-ray wavelength (0.154184 nm), θ is the Bragg angle, and β is the peak broadening, determined from the full width at half-maximum (FWHM) of the diffraction peaks. The results from these calculations are shown in Table 1.

The morphological features of carbonated HA NPs were characterized by Transmission Electron Microscopy (TEM) using a FEI TECNAI G^2^ F20 microscope operating at 200 kV. Raman spectroscopy was performed using an inVia confocal Raman microscope (Renishaw, U.K.) equipped with a 532 nm laser (50% power), a 10× objective, and a grating of 1800 lines/mm. Spectra were collected over the 350–1200 cm^–1^ range with an acquisition time of 10 s and 5 accumulations. Fourier-transform infrared spectroscopy (FTIR) was performed using a Nicolet iS50 spectrometer (Thermo Scientific) in absorbance mode, with sample preparation based on the KBr pellet method. Spectra were acquired in the range of 500–4000 cm^–1^, with a resolution of 4 cm^–1^ and 64 scans per measurement. Data were collected in transmittance mode and automatically converted to absorbance, followed by baseline correction and normalization to the ν_3_PO_4_ band with the highest absorbance using the OMNIC 9 software.

The carbonate content (*w*(CO_3_)) in each sample was estimated based on the carbonate-to-phosphate ratio (*r*_c/p_), calculated from the integrated areas of the ν_3_CO_3_ and ν_1_,ν_3_PO_4_ bands observed in the FTIR spectra using the method described by Grunenwald et al.^50^ The Ca and P weight percentages (*w*(Ca) and *w*(P), respectively) were estimated using inductively coupled plasma-optical emission spectroscopy (ICP-OES) with a PerkinElmer Avio 550 Max analyzer. The mass loss during thermal treatment was evaluated by thermogravimetric analysis (TGA) using a TA Instruments SDT-Q600 system, with ∼5 mg of NPs placed in an alumina crucible for each sample. The analysis was performed from 25 to 980 °C at a heating rate of 10 °C/min under a flow of synthetic air. Photoluminescence emission spectra of as-synthesized and heat-treated carbonated HA NPs were acquired using a Horiba Jobin Yvon Fluorolog-3 spectrofluorometer equipped with a Hamamatsu R928 photomultiplier tube. A 450 W continuous-wave xenon arc lamp served as the excitation source. Measurements were performed at λ_exc_ = 405, 488, 561, and 640 nm. All emission intensities were recorded by photon counting and corrected for the spectral response of the system.

### 2.4 Citrate Functionalization

The HAS8–450 NPs were selected for the biological assays due to their optimized photoluminescence performance. To this end, the NPs were functionalized with citrate ions to improve their colloidal stability and dispersion, following the protocol previously established in our study on carbonated ACP NPs.^48^ Briefly, the HAS8–450 NPs were dispersed in an aqueous trisodium citrate solution (Na_3_C_6_H_5_O_7_, Sigma-Aldrich) prepared at 24 mM and pH 7.4, to obtain a final concentration of 0.5 mg mL^–1^ based on the weighed nanoparticle powder mass. The resulting suspension was sonicated in an ultrasonic bath at 100% power for 10 min and further stirred for 3 h. Subsequently, the sample was thoroughly purified by centrifugation at 12,000 rpm for 10 min with ultrapure water. The final pellet, consisting of citrate-functionalized HAS8–450 NPs (hereafter referred to as HAS8–450-Cit NPs), was resuspended in water to obtain a stock suspension at 2.5 mg mL^–1^, based on the initially weighed nanoparticle mass. For comparison, the same procedure was carried out for HAS8–450 NPs using ultrapure water instead of trisodium citrate solution.

The hydrodynamic diameter and zeta potential (ZP) of HAS8–450 and HAS8–450-Cit NPs were measured by dynamic light scattering (DLS) and electrophoretic mobility, respectively, using a Zetasizer Nano ZS (Malvern Instruments). Measurements were performed at 633 nm and 90° detection angle in water by diluting the stock suspensions at a concentration of 100 μg mL^–1^. The photoluminescence emission spectra of HAS8–450 and HAS8–450-Cit NPs were recorded in diluted aqueous suspensions at 1 mg mL^–1^ using a Horiba Jobin Yvon Fluorolog-3 spectrofluorometer at the excitation wavelength used in the biological assays (λ_exc_ = 488 nm).

### 2.5 *In Vitro* Assays

Cytotoxicity was assessed via MTT assay using human neonatal dermal fibroblast (HDFn) cells cultured in DMEM (Dulbecco’s Modified Eagle Medium) supplemented with 10% FBS (Fetal Bovine Serum) at 37 °C and 5% CO_2_. Cells were seeded in 96-well plates at 4 × 10^4^ cells mL^–1^ and, after 24 h, treated with HAS8–450-Cit NPs at concentrations of 10, 20, 40, 80, 160, and 320 μg mL^–1^ for 24 h. The tested concentrations were obtained by serial dilution in complete medium from the stock suspension at 2.5 mg mL^–1^. After incubation, cells were exposed to 3-[4,5-dimethylthiazol-2-yl]-2,5 diphenyltetrazolium bromide (MTT) solution (0.5 mg mL^–1^) for 1 h. The resulting formazan crystals were solubilized in dimethyl sulfoxide, and absorbance was recorded at 570 nm using a SpectraMax M3 microplate reader. Cell viability was calculated relative to untreated controls. For flow cytometry analysis, HDFn cells were seeded in 12-well plates at 8 × 10^4^ cells mL^–1^ and incubated for 24 h, followed by treatment for 4 or 24 h with HAS8–450-Cit NPs diluted in complete medium at 320 μg mL^–1^, prepared from the 2.5 mg mL^–1^ stock suspension. After incubation, cells were washed with PBS, detached using trypsin-EDTA, centrifuged, and resuspended in sheath fluid for analysis on a FACSCalibur flow cytometer (BD Biosciences), recording 10000 events per sample. Data were processed using FlowJo software. Untreated cells served as the negative control. These experiments were conducted in triplicate across three independent trials, and results were analyzed by one-way (MTT) or two-way (flow cytometry) ANOVA with Tukey’s multiple comparisons test.

Cellular bioimaging was evaluated using confocal laser scanning microscopy (CLSM). HDFn cells were seeded at 1 × 10^5^ cells mL^–1^ on round glass coverslips placed in 24-well plates. After 24 h incubation, cells were treated with HAS8–450-Cit NPs suspended in complete medium at 320 μg mL^–1^, prepared from the 2.5 mg mL^–1^ stock suspension, and maintained for an additional 4 h. Cells were fixed with 4% paraformaldehyde, stained with CellMask Deep Red and Hoechst 33342. Coverslips were mounted on glass slides and dried for 24 h. Imaging was performed using a Zeiss LSM900 inverted confocal microscope.

## 3 Results and Discussion

### 3.1 Structural and Morphological Analysis

Figure 1 shows the XRD patterns of the as-synthesized HAS1 to HAS8 samples. The diffraction peaks observed are consistent with the poorly crystalline hexagonal phase of HA, in agreement with the ICSD card No. 26204.^51^ The absence of peaks corresponding to secondary or deleterious phases confirms the successful synthesis of single-phase HA under the conditions employed. A progressive broadening and reduced resolution of the diffraction peaks can be observed with increasing [CO_3_^2–^]/[PO_4_^3–^] molar ratios, particularly evident in samples HAS5 to HAS8. This well-documented trend indicates a response to the incorporation of CO_3_^2–^ into HA lattice, characterized by a decrease in its long-range structural ordering and a reduction in crystal size.^29^ To gain deeper insight into this behavior, the crystallite sizes of the NPs were estimated through the *D*_002_ and *D*_300_ values, as presented in Table 1. A clear reduction tendency in crystallite sizes is observed when progressing from sample HAS1 to HAS8, with *D*_002_ and *D*_300_ decreasing from 41 and 19 nm in HAS1 to 22 and 14 nm in HAS8, respectively. Additionally, the *D*_002_/*D*_300_ ratios decrease in samples with higher carbonate content, indicating reduced anisotropy in the crystallites.

**Figure 1:**
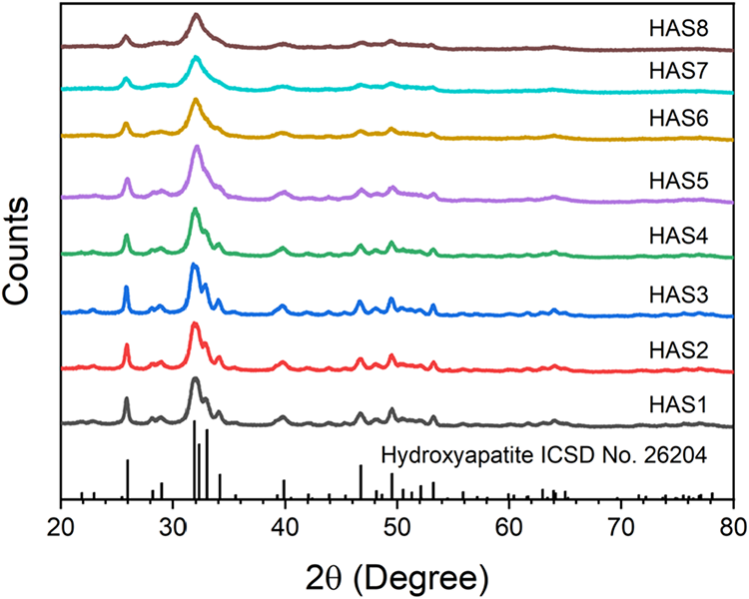
XRD patterns of the HA NPs prepared by chemical precipitation at RT. Samples labeled HAS1 to HAS8 correspond to materials synthesized using different [CO_3_^2–^]/[PO_4_^3–^] molar ratios, with the specific values and crystallite size results summarized in Table 1.

TEM analysis of representative as-synthesized samples with low (HAS1 and HAS4) and high (HAS5 and HAS8) carbonate contents, shown in Figure 2a–d and complemented by additional micrographs available in Figure S1, confirms that all samples consist of nanorods. As the [CO_3_^2–^]/[PO_4_^3–^] molar ratio increased, the Selected Area Electron Diffraction (SAED) patterns evolved from spot-like halos to more continuous rings, indicating a reduction in crystallinity. Moreover, the NPs exhibited a decrease in both length and width from sample HAS1 to HAS8, as determined from size measurements of 100 particles per sample using ImageJ software, performed on suitable regions from at least 15 TEM micrographs available for each case. The average length decreased from 30 ± 6 to 16 ± 3 nm and the average width from 15 ± 3 to 9 ± 2 nm for samples HAS1 and HAS8, respectively. These findings are consistent with the XRD data and demonstrate that CO_3_^2–^ incorporation reduces the long-range structural order of HA and inhibits crystal growth, especially in the highly carbonated samples. After thermal treatment, as illustrated for samples HAS8–400 (17 ± 3 × 10 ± 2 nm) and HAS8–450 (18 ± 3 × 10 ± 2 nm) in Figure S2a,b, the particle sizes remained essentially unchanged compared with those of the as-synthesized HAS8 NPs (16 ± 3 × 9 ± 2 nm). Only a slight improvement in particle definition and morphology was observed, as also supported by the complementary TEM images shown in Figure S1. Although the SAED patterns of these samples show no significant changes (Figure S2), the subsequent FTIR and TGA analyses revealed an increase in structural and surface ordering along with the removal of volatile species, such as adsorbed and structural water, as a result of heat treatment. These effects possibly contribute to the formation of more uniform and well-defined nanocrystals.

**Figure 2:**
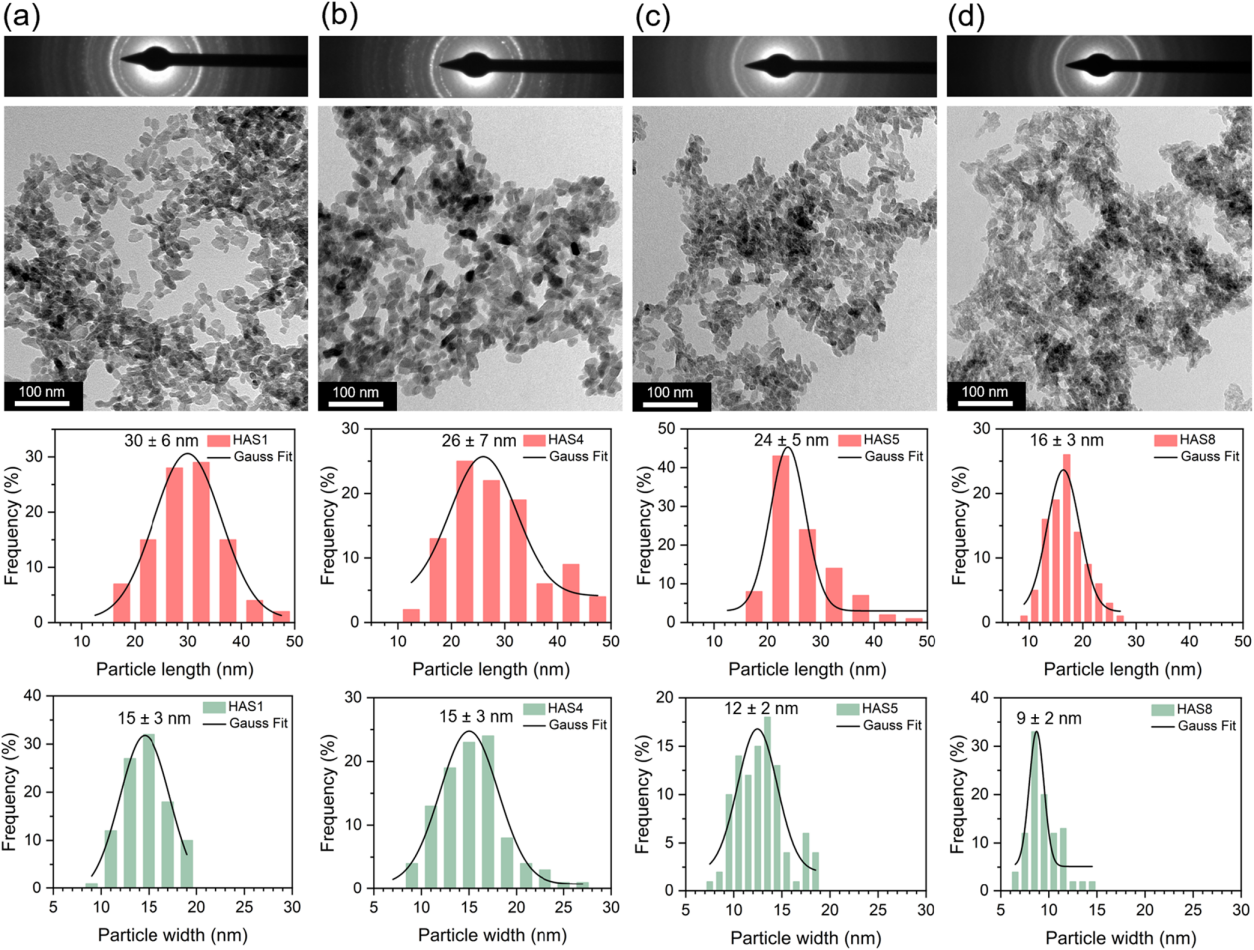
TEM analysis of the as-synthesized carbonated HA NPs: (a) HAS1, (b) HAS4, (c) HAS5, and (d) HAS8.

Figure 3a–c presents the FTIR spectra of as-synthesized carbonated HA samples and those heat-treated at 400 and 450 °C, while Figure 3d–f shows the expanded region from 1800 to 1300 cm^–1^. The characteristic vibrational modes associated with distorted PO_4_^3–^ tetrahedra in HA structure are observed in all samples: the bands at 567 and 603 cm^–1^ correspond to the O–P–O bending mode (ν_4_PO_4_), the band at 960 cm^–1^ to the symmetric stretching of P–O (ν_1_PO_4_), and the bands at 1036 and 1095 cm^–1^ to the asymmetric stretching of P–O (ν_3_PO_4_).^52^ These results indicate that the HA structure remained stable under the heat treatment conditions used. As expected, bands attributed to CO_3_^2–^ groups incorporated into the HA lattice are also present in as-synthesized and thermally treated samples. This includes the HAS1 sample, prepared without the CO_3_^2–^ precursor, where the carbonate incorporation originated from trace amounts present in the water or from atmospheric CO_2_ dissolution.^53^ The observed bands involve the out-of-plane bending mode of O–C–O (ν_2_CO_3_) around 860–880 cm^–1^ and the asymmetric stretching of C–O (ν_3_CO_3_) in the 1570–1340 cm^–1^ region.^54^ The intensity of these bands increases with the initial [CO_3_^2–^]/[PO_4_^3–^] molar ratio, indicating greater CO_3_^2–^ incorporation into the HA structure. As shown in Figure S3, this trend was further corroborated by collecting the Raman spectra for the as-synthesized NPs. The decrease in the intensity of these bands in the heat-treated samples, compared with the corresponding as-synthesized NPs, is associated with partial decarbonation of the structure resulting from the irreversible thermal decomposition of CO_3_^2–^ groups.^37^ This process may occur through reactions such as^38^

2 $${\mathrm{CO}}_{3}^{2-}+H_{2}O\to {\mathrm{OH}}^{-}+{\mathrm{CO}}_{2}↑$$

The bands at 3568 and 634 cm^–1^, attributed to the lattice OH^–^ groups in HA,^52^ are detectable in samples with low CO_3_^2–^ content (HAS1-HAS4) and become progressively less intense with increasing CO_3_^2–^ incorporation. This is consistent with the depletion of OH^–^ from the HA lattice as CO_3_^2–^ incorporation increases, a process that promotes the formation of hydroxyl vacancies (V_OH_) vacancies through the substitution mechanisms discussed below. In samples with the highest CO_3_^2–^ contents (HAS5-HAS8), the OH^–^ bands are no longer detectable due to the high density of V_OH_ vacancies. The same trends are maintained in the heat-treated groups at 400 and 450 °C, meaning that the samples with higher CO_3_^2–^ levels still exhibit lower densities of OH^–^ groups. However, small shoulders at 3568 and 634 cm^–1^ appear in the heat-treated samples, particularly in HAS5-HAS7, indicating partial rehydroxylation within the hexagonal sites. This behavior is mainly attributed to the thermally induced decomposition of CO_3_^2–^ groups, as represented by eq 2.^38^ Furthermore, bands between 3700 and 3000 cm^–1^ and at 1640 cm^–1^, attributed to adsorbed and structural H_2_O, decrease in intensity upon thermal treatment. This decrease is mainly associated with the irreversible elimination of structural H_2_O molecules within HA lattice, since the loss of adsorbed H_2_O is a reversible process.^41^

**Figure 3:**
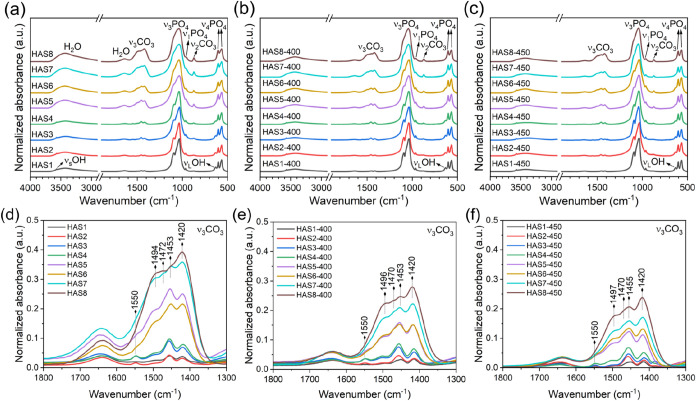
FTIR spectra of purified carbonated HA samples: (a) as-synthesized, (b) heat treated at 400 °C, and (c) heat treated at 450 °C. The region corresponding to the ν_3_CO_3_ bands from 1800 to 1500 cm^–1^ is shown in (d) for as-synthesized samples, (e) for samples treated at 400 °C, and (f) for samples treated at 450 °C.

FTIR spectra can provide insights into the sites occupied by CO_3_^2–^ groups within the HA lattice, as the positions of the ν_2_CO_3_ and ν_3_CO_3_ bands vary depending on the substitution mechanism.^55^ In type A substitution, one CO_3_^2–^ group replaces an OH^–^ group and generates a V_OH_ vacancy in the hexagonal channel to maintain charge balance, without altering the Ca/P ratio

$$2{OH}^{-}\to {CO}_{3}^{2-}+V_{OH}\;\left[Type\;A\right]$$ 3

Type B substitution involves the replacement of PO_4_^3–^ groups and may occur via different mechanisms.^29^ In one case, two CO_3_^2–^ groups replace two PO_4_^3–^ groups, generating one calcium vacancy (V_Ca_) and increasing the Ca/P molar ratio

$${\mathrm{Ca}}^{2+}+{2\mathrm{PO}}_{4}^{3-}\to {2\mathrm{CO}}_{3}^{2-}+V_{\mathrm{Ca}}\;\left[\mathrm{Type}\;B1\right]$$ 4

In the other case, the substitution of PO_4_^3–^ by CO_3_^2–^ occurs through the formation of V_OH_ and V_Ca_ vacancies, without changing the Ca/P molar ratio

$${\mathrm{Ca}}^{2+}+{\mathrm{PO}}_{4}^{3-}+{\mathrm{OH}}^{-}\to {\mathrm{CO}}_{3}^{2-}+V_{\mathrm{Ca}}+V_{\mathrm{OH}}\;\left[\mathrm{Type}\;B2\right]$$ 5

There is no consensus regarding the dominant mechanism for B-type substitution, which is believed to occur through a combination of pathways and may involve additional counterions such as Na^+^ and NH_4_^+^, depending on synthesis conditions.^31,56^ Furthermore, oxygen vacancies (V_O_) within PO_4_^3–^ tetrahedra resulting from B-type substitution were also identified.^39^ Although B-type substitution is generally more prevalent, HA samples containing CO_3_^2–^ frequently exhibit both mechanisms, known as AB-type.^54^

In this study, the main ν_3_CO_3_ components observed in the as-synthesized samples and preserved after heat treatment (Figure 3d–f) indicate that the materials correspond to AB-type substituted HA.^57,58^ This interpretation is supported by the deconvolution of the ν_2_CO_3_ vibrational mode shown in Figure S4. Multiple components within this band indicate distinct local environments, with three components consistently detected in all samples. The band at approximately 873 cm^–1^ has been associated with B-type substitution, while the bands at around 864 and 879 cm^–1^ have been assigned to A-type substitution in two distinct orientations of CO_3_^2–^ within the hexagonal channels.^59^ Following the approach detailed by Fleet,^60^ the ratio ν_2_CO_3_(B)/ν_2_CO_3_(A), obtained from the integrated areas of the ν_2_CO_3_ deconvoluted bands, provides insight into the preferential substitution. As shown in Figure 4a, A-type substitution dominates in the low-carbonated samples (HAS1-HAS4), whereas B-type becomes progressively more prominent in the higher-carbonated samples (HAS5-HAS8), a trend that is consistent with the literature.^55^ Although CO_3_^2–^ decomposition at the temperatures used is mainly associated with A-type species,^38^ the decrease in ν_2_CO_3_(B)/ν_2_CO_3_(A) ratio after heating the highly carbonated samples (HAS5-HAS8) indicates a relative increase of A-type substitution from the as-synthesized state to 400 and 450 °C. According to Ivanova et al.,^39^ this behavior results from a thermally induced rearrangement of CO_3_^2–^ from tetrahedral to hexagonal sites, partially compensating A-type decarbonation. Nevertheless, even after this reorganization, B-type substitution remains predominant in these heat-treated samples except HAS5–450.

**Figure 4:**
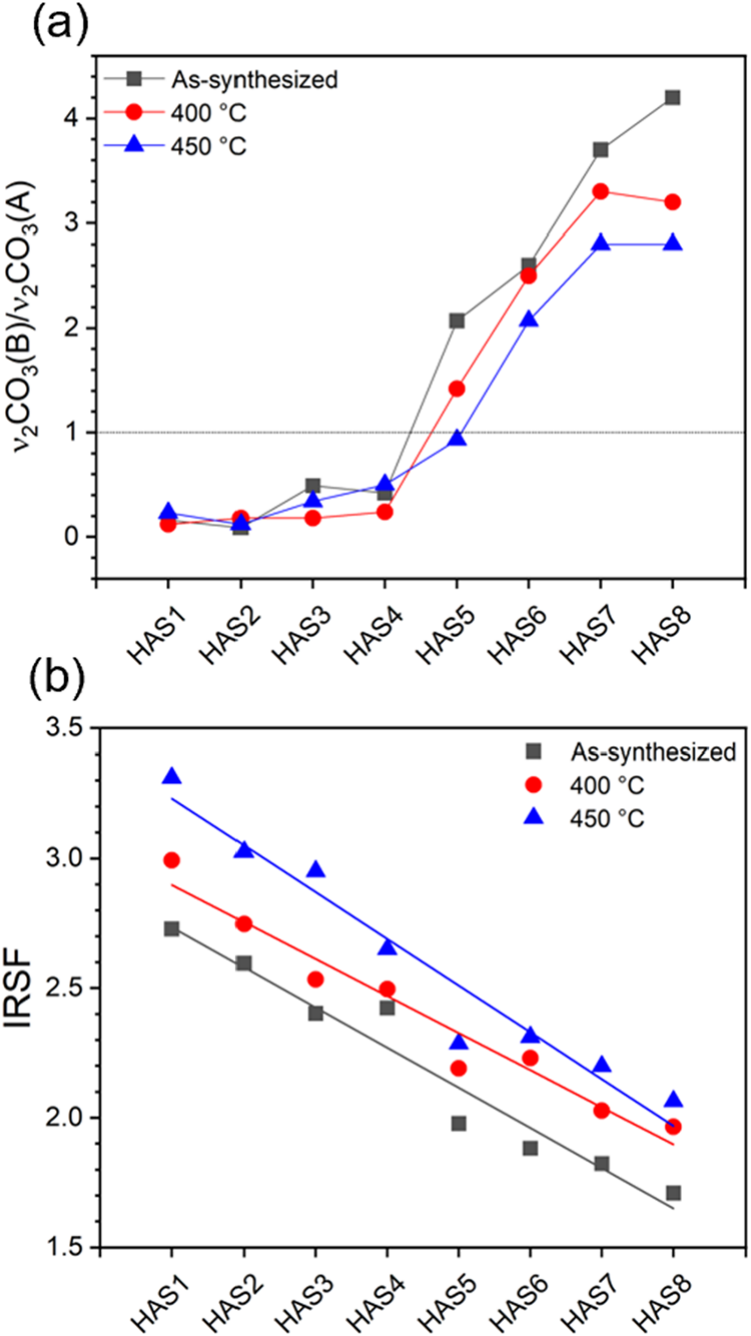
Calculated parameters from the FTIR spectra of carbonated HA NPs before and after heat treatment at 400 or 450 °C. (a) Ratios between the integrated areas of the individual components obtained from the deconvolution of the ν_2_CO_3_ band, which serve as an indicative of a preferential A-type or B-type CO_3_^2–^ substitution (mean uncertainty: ±0.13), and (b) short-range structural order in carbonated HA NPs, assessed using the IRSF parameter derived from the splitting of the ν_4_PO_4_ bands (mean uncertainty: ±0.12).

In the FTIR spectra, the ν_3_PO_4_ and ν_4_PO_4_ bands become progressively broader and less defined with increasing CO_3_^2–^ content in both as-synthesized and thermally treated samples (Figure 3a–c). Similar trends are observed in the Raman spectra (Figure S3). In accordance with previous studies, these features are attributed to a wider distribution of vibrational environments within PO_4_^3–^ groups, which reflects fluctuations in P–O bond lengths and O–P–O angles, i.e., increased short-range structural disorder as a function of CO_3_^2–^ incorporation.^61,62^ To complement the qualitative analysis of band amplitudes, the relative short-range order was assessed in terms of the infrared splitting factor (IRSF), defined as the FTIR ratio (Ab_603_ + Ab_567_)/Ab_590_ originally proposed by Shemesh.^63^ Ab_603_ and Ab_567_ represent the absorbance values of ν_4_PO_4_ bands at 603 and 567 cm^–1^, and Ab_590_ corresponds to the absorbance at 590 cm^–1^, in the minimum between these two bands. This ratio reflects the degree of local distortion within the ν_4_PO_4_ bending region and correlates well with variations in structural ordering and CO_3_^2–^ content across different samples.^64^

As shown in Figure 4b, the IRSF progressively decreases with increased CO_3_^2–^ content, confirming the loss of short-range structural order. The IRSF values reduce from 2.7 to 1.7 in as-synthesized samples, from 3.0 to 2.0 in samples treated at 400 °C, and from 3.3 to 2.1 at 450 °C. This behavior results from enhanced heterogeneity in PO_4_^3–^ bonds, associated with higher density of crystal defects, including CO_3_^2–^ substitution, V_Ca_, V_OH_, and V_O_ (within PO_4_^3–^ sites) vacancies, as well as distorted lattice sites.^60^ In addition, the observed reduction in crystal size increases the proportion of surface-exposed PO_4_^3–^ groups, where surface relaxation and under-coordinated sites generate more disordered local environments, further contributing to the broadening of the ν_4_PO_4_ modes and the decrease in IRSF.^65^ Thermally treated samples show higher IRSF values compared to their as-synthesized counterparts, consistent with thermally induced increase in lattice and surface ordering.^66^

### 3.2 Compositional Analysis

The *w*(Ca) and *w*(P) contents of the as-synthesized carbonated HA NPs were determined by ICP-OES analysis, enabling the calculation of their respective Ca/P molar ratios. As shown in Figure 5a, the Ca/P values for samples HAS1 to HAS5 were lower than that of stoichiometric HA (Ca/P = 1.667), a behavior well documented in the literature due to the formation of calcium-deficient HA under the synthesis conditions employed.^46^ In this case, V_Ca_ vacancies are associated with the substitution of PO_4_^3–^ by acidic phosphate groups (HPO_4_^2–^), which also leads to the formation of additional V_OH_ vacancies. Moreover, a continuous increase in the Ca/P molar ratio was observed up to sample HAS8, reaching values higher than that of stoichiometric HA. This behavior is attributed to the progressive incorporation of CO_3_^2–^ via B1-type substitution, as described in eq 4, where two phosphate groups are replaced by two carbonate groups, resulting in the formation of V_Ca_ to maintain structural charge balance.

**Figure 5:**
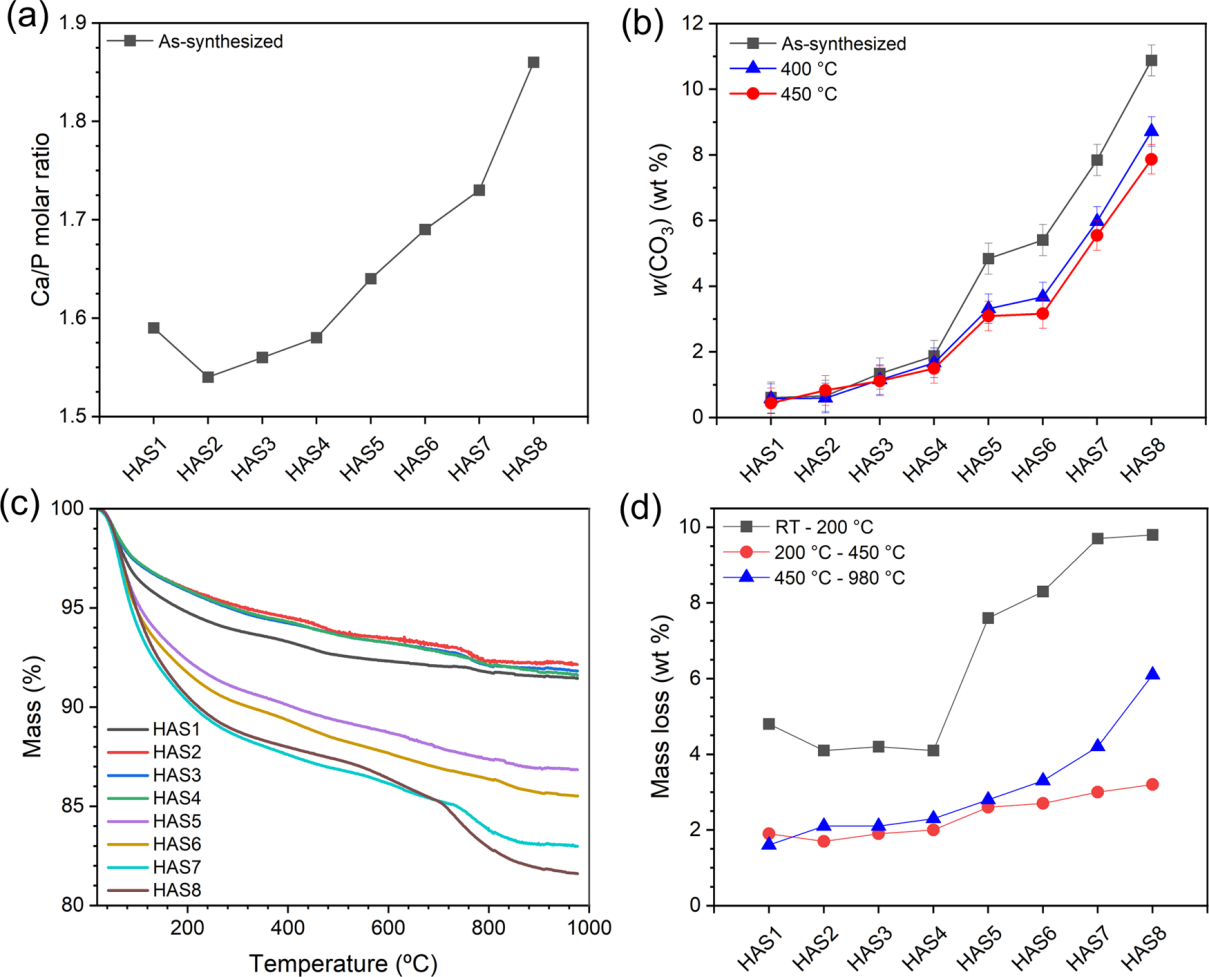
Compositional analysis of the carbonated HA samples: (a) Ca/P molar ratio of the as-synthesized NPs estimated by ICP-OES, (b) CO_3_^2–^ content (*w*(CO_3_)) determined by FTIR for all samples, (c) TGA curves of the as-synthesized samples, and (d) corresponding mass losses in distinct temperature ranges.

The FTIR-derived *w*(CO_3_) values are presented in Figure 5b and Table S1. A gradual increase in CO_3_^2–^ content is observed for both as-synthesized and thermally treated sample groups, consistent with the trend observed in the Ca/P molar ratio. In the as-synthesized samples, *w*(CO_3_) ranges from 0.6 wt % in HAS1 to 10.9 wt % in HAS8, which corroborates the findings of Deymier et al.^67^ for HA samples prepared with similar [CO_3_^2–^]/[PO_4_^3–^] molar ratios. Thermal treatment led to a reduction in *w*(CO_3_) compared to the corresponding as-prepared samples, particularly at higher initial CO_3_^2–^ concentrations due to partial decarboxylation of HA lattice. For samples treated at 400 °C, *w*(CO_3_) values range from 0.6 wt % (HAS1–400) to 8.7 wt % (HAS8–400), while for those treated at 450 °C, the values range from 0.4 wt % (HAS1–450) to 7.9 wt % (HAS8–450).

Figure 5c shows the TGA curves of the as-synthesized HA samples. The TGA curves were divided into three main regions, and the corresponding mass losses for area are illustrated in Figure 5d. The first region extends from RT to 200 °C and is associated with the release of adsorbed water.^37^ In this interval, mass loss initially ranged from 4.8 to 4.1 wt % for samples HAS1 to HAS4, followed by a progressive increase to values between 7.6 and 9.8 wt % for samples HAS5 to HAS8. This trend can be explained by the reduction in crystal size observed in our samples by XRD and TEM with increasing carbonate content, which leads to a higher specific surface area and, consequently, enhances water adsorption on the surface of the NPs.^68^ Moreover, CO_3_^2–^ incorporation has been shown to increase the water-binding surface energy of HA NPs, which may contribute to its greater affinity for water adsorption.^69^

The second region of mass loss, from 200 to 450 °C, corresponds to the highest heat treatment temperature employed in this study and is mainly associated with the elimination of structural water and entrapped NH_4_^+^ ions.^41^ The loss of structural water observed by TGA is consistent with the FTIR results, which showed the attenuation of bands associated with these species. Structural water molecules were reported to mainly occupy the hexagonal channels of the HA structure.^60^ Their presence is favored by the formation of V_OH_, either resulting from A-type and B2-type CO_3_^2–^ substitutions or from charge compensation associated with HPO_4_^2–^ groups, both of which are present in our study. Furthermore, structural water has also been associated with V_O_ in PO_4_^3–^, resulting from B-type carbonates and may even occupy V_Ca_ sites.^39^

A progressive increase in mass loss was observed in this region from 1.9 wt % in sample HAS1, which has the lowest CO_3_^2–^ content, to 3.2 wt % in sample HAS8, which contains the highest concentration of CO_3_^2–^. This increment can be attributed to the progressive structural disorder of the carbonated HA lattice, which promotes the entrapment of a slightly higher amount of water molecules within defect sites, particularly in association with the ionic vacancies. Additionally, this increase can also be related to the partial release of CO_3_^2–^ as CO_2_. The release is likely due to the partial removal of A-type carbonate groups (eq 2), and possible decomposition reactions involving these groups and HPO_4_^2–^ with further release of water.^38,39^ This interpretation is consistent with the variations in FTIR-derived *w*(CO_3_) values between the as-synthesized and heat-treated samples, confirming the partial decomposition of CO_3_^2–^ at these temperatures alongside the release of structural water.

Finally, the third mass loss region (450–980 °C) is attributed to further decarbonation of the HA structure, involving the decomposition of both A-type and B-type carbonates.^37,38^ The mass loss in this region increased significantly from 1.5 wt.% in sample HAS1 to 6.1 wt.% in HAS8, in agreement with the progressively higher CO_3_^2–^ content in these samples. This result confirms that a substantial fraction of CO_3_^2–^ remains incorporated within the HA structure and only undergoes more pronounced release at temperatures higher than those employed in this study. Moreover, this region also includes the release of H_2_O resulting from both the condensation of HPO_4_^2–^ groups into P_2_O_7_^4–^ and the subsequent decomposition of P_2_O_7_^4–^ into PO_4_^3–^.^38^

### 3.3 Photoluminescence of Chemically Precipitated HA NPs

The as-synthesized and heat-treated carbonated HA NPs were characterized for their photoluminescent properties. The excitation wavelength of 405 nm was selected based on previous literature.^22^ Figure 6a presents the emission spectra obtained for the as-synthesized HA NPs, which feature two main emission maxima at 438 nm (2.83 eV) and 465 nm (2.67 eV), with additional shoulders around 500 nm (2.48 eV) and 540 nm (2.30 eV). A graph with a magnified *y*-axis scale showing the emission spectra of all as-synthesized samples is presented in Figure S5. The components of the emission profile become more evident upon spectral deconvolution, as illustrated in Figure 6b. Among these, the emission at 438 nm is the most intense in all samples. The observed photoluminescence originates from the recombination of e^–^–h^+^ pairs involving energy states located within the forbidden zone.^23^

**Figure 6:**
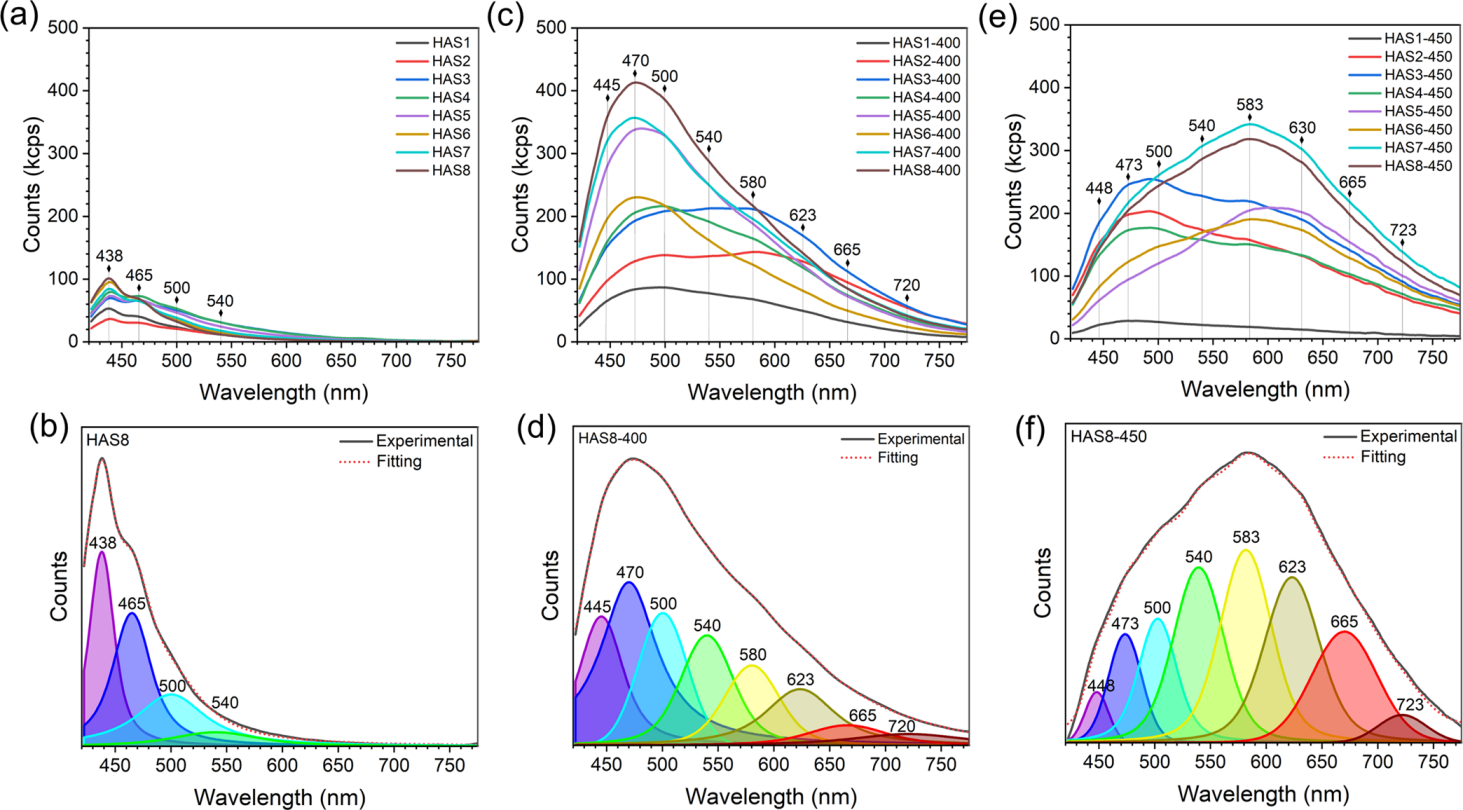
Photoluminescence properties of carbonated HA NPs at λ_exc_ = 405 nm: (a) Emission spectra of the as-synthesized samples, (b) spectral deconvolution of the HAS8 emission profile, (c) emission spectra of samples heat-treated at 400 °C, (d) spectral deconvolution of the HAS8–400 emission profile, (e) emission spectra of samples heat-treated at 450 °C, and (f) spectral deconvolution of the HAS8–450 emission profile.

The emission bands observed for samples HAS1 to HAS8 closely resemble those previously reported for HA and calcium-deficient HA synthesized by chemical precipitation at 90 °C,^23^ HA obtained via hydrothermal treatment,^24^ and for carbonated ACP obtained by chemical precipitation.^48,70^ This spectral similarity indicates that analogous intrinsic defect-related states govern the photoluminescence in all these calcium phosphate materials. Importantly, the increase in emission intensity observed from HAS1 to HAS8 suggests that the luminescence is predominantly controlled by the density of these emissive centers, which is in turn modulated by the extent of CO_3_^2–^ incorporation into the HA lattice.

Consistent with the structural analyses, the increasing incorporation of CO_3_^2–^ leads to enhanced structural disorder at both short and long ranges, as well as at the NPs surfaces. Photoluminescence, which is highly sensitive to local atomic order, reflects these structural changes in a direct and complementary manner, with higher emission intensities corresponding to increased lattice disorder.^71^ We propose that this disorder affects the photoluminescence behavior of our carbonated HA through two main mechanisms. First, the increased local distortion of the PO_4_^3–^ tetrahedra detected by structural characterization and, consequently, of the calcium polyhedra increases the population of optically active shallow defect levels, i.e., those located near the band edges, by broadening the distribution of Ca–O and P–O bond lengths and angles.^23^ Second, substitutional incorporation of CO_3_^2–^ promotes the formation of a higher concentration of V_OH_ vacancies through A-type substitution, as well as V_Ca_, V_OH_, and V_O_ within PO_4_^3–^ tetrahedra in the case of B-type substitution. While V_Ca_ vacancies have been associated with optically active shallow defect levels,^23^ V_OH_ and V_O_ (in PO_4_^3–^) vacancies were linked to deeper states within the band gap, i.e., toward the middle of the bandgap.^24,25^ These effects occur concomitantly as a result of CO_3_^2–^ incorporation and collectively contribute to the enhancement of photoluminescence intensity in precipitated HA.

Comparison with ACP further clarifies these findings. In a previous study, we demonstrated notable similarities between HA and ACP emission profiles.^70^ More recently, we showed that the incorporation of CO_3_^2–^ into ACP markedly enhances the photoluminescence intensity, reaching a maximum at approximately 11.1 wt % of CO_3_^2–^.^48^ In the present study, carbonated HA displayed the same trend, with emission intensity progressively increasing up to 10.9 wt %. However, the magnitude of the photoluminescence enhancement in ACP is significantly higher than in HA at comparable CO_3_^2–^ levels. It is important to note that in both studies the photoluminescence emission spectra were recorded under the same experimental conditions.

Two key structural aspects appear to account for this difference. First, the crystalline nature of HA imposes intrinsic constraints on structural distortion and defect formation, limiting the density of optically active centers compared to the inherently disordered amorphous lattice of ACP. As a result, the generation of shallow defect states associated with distortions in Ca–O and P–O bond lengths and angles is more restricted in HA, particularly at lower levels of CO_3_^2–^ incorporation. Second, while PO_4_^3–^ → CO_3_^2–^ substitution dominates at all CO_3_^2–^ levels in ACP, HA exhibits a mixed AB-type substitutional behavior. At lower CO_3_^2–^ contents, A-type substitution (replacement of OH^–^) prevails, whereas B-type substitution (replacement of PO_4_^3–^) becomes more prominent at higher levels. This suggests that the density of optically active defects arising from phosphate substitution tends to be lower in HA than in ACP under comparable CO_3_^2–^ contents. Taken together, these findings indicate that both the local distortions induced by increasing CO_3_^2–^ incorporation and the progressive increment in the concentration of vacancies, particularly those arising from B-type substitution, are crucial in modulating the photoluminescence of HA. From a broader perspective, PO_4_^3–^ → CO_3_^2–^ substitution can be assumed to play critical role in the photoluminescence of calcium phosphate materials.

Further comparison with calcium-deficient HA reinforces this interpretation. In our previous study,^23^ no significant enhancement in photoluminescence intensity was observed in calcium-deficient HA in comparison to more stoichiometric HA, despite the expected increase in V_Ca_ and V_OH_ densities. This further highlights the role of B-type substitution in enhancing the photoluminescent properties of HA by increasing atomic disorder, mainly through lattice distortions, the formation of V_O_ vacancies within PO_4_^3–^ tetrahedra in association with V_Ca_ and V_OH_ vacancies, as well as through additional surface disorder resulting from the reduction in crystal size. Nevertheless, the possible contribution of defect-related energy levels directly introduced by CO_3_^2–^ impurities to the photoluminescent emission cannot be discarded. Overall, the results confirm that CO_3_^2–^ incorporation emerges as a central factor influencing the photoluminescence of HA NPs synthesized via wet chemical routes. By modulating structural disorder and vacancy populations, CO_3_^2–^ substitution defines the density of emissive defects states, thereby governing the photoluminescence properties.

### 3.4 Photoluminescence of Heat-Treated HA NPs

Figure 6c shows the emission spectra of HA NPs subjected to thermal treatment at 400 °C. The emission components observed for the as-synthesized samples are retained after thermal treatment. However, except for samples HAS2–400 and HAS3–400, the emission component at 470 nm becomes the most intense. Particularly, the emission bands exhibit pronounced broadening, now spanning almost the entire visible region of the electromagnetic spectrum. This behavior is attributed to the emergence of additional emissive centers at 580 nm (2.14 eV), 623 nm (1.99 eV), 665 nm (1.86 eV), and 720 nm (1.72 eV) in all heat-treated samples, as more clearly evidenced by the deconvoluted spectrum of the HAS8–400 sample shown in Figure 6d. tensity of the thermally treated samples increases by approximately 2- to 5-fold relative to their as-synthesized counterparts. A comparison of the HAS1–400 to HAS8–400 samples shows that this intensity enhancement results in a trend similar to that observed in the as-synthesized series, in which increasing CO_3_^2–^ incorporation during synthesis correlates with higher luminescence, particularly when B-type substitution becomes dominant (HAS5–400 to HAS8–400). Notably, HAS8–400 exhibits the most intense emission among all samples.

Figure 6e presents the emission spectra of samples thermally treated at 450 °C. In these samples, both the number and positions of the emission components remain similar to those observed for the samples treated at 400 °C. A decrease in emission intensity is particularly evident for sample HAS1–450 when compared to HAS1–400 and to the original as-synthesized HAS1, which contained the lowest amount of CO_3_^2–^. For the following samples with low CO_3_^2–^ content (HAS2–450 to HAS4–450) which are mainly dominated by A-type CO_3_^2–^ substitution, the emission intensities and spectral profiles remain relatively comparable to their counterparts treated at 400 °C, showing only variations in the relative intensities of individual emission components. In contrast, the most pronounced spectral changes are observed in samples with higher CO_3_^2–^ contents (HAS5–450 to HAS8–450), where B-type CO_3_^2–^ substitution dominates and the lower-energy emission components in the orange-to-red region (540, 583, 623, and 665 nm) become significantly more intense (Figure 6f). In these samples, the emission maximum shifts toward 583 nm, accompanied by a slight reduction in intensity relative to the respective 400 °C-treated samples. Among all samples treated at 450 °C, those with the highest CO_3_^2–^ content (HAS7–450 and HAS8–450) exhibit the most intense photoluminescence, reinforcing the trend observed in both the samples treated at 400 °C and the as-synthesized series. Moreover, the heat-treated samples, particularly those with higher CO_3_^2–^ content and treated at 450 °C, also exhibited the highest emission intensities when excited at shorter wavelengths (λ_exc_ = 488, 561, and 640 nm), i.e., lower energies (Figure S6).

Together, these results demonstrate that both the emission intensity and the spectral profile of HA strongly dependent on the amount of CO_3_^2–^ incorporation, its dominant substitution type, and the heat treatment temperature. Notably, the HAS1 sample exhibited enhanced emission intensity and a broadening of the emission profile following thermal treatment at 400 °C. However, a pronounced decrease in intensity was observed at 450 °C. This behavior is attributed to a combination of increased lattice ordering and the partial removal of CO_3_^2–^, which reduce the population of radiative defect-related energy states.^41^ In contrast, this thermally induced quenching effect on emission intensity, which started at 450 °C, is retarded in all other samples. This phenomenon is likely associated with the greater CO_3_^2–^ incorporation in the as-synthesized samples and the increased retention of these groups within the HA lattice at 400 to 450 °C, which contributes to the stabilization of higher structural and surface disorder at these temperatures.

Due to the complex defective structure of HA, energy levels located near both the conduction and valence bands, such as those associated with distortions in Ca–O and P–O bonds, V_Ca_, and V_O_ in OH^–^, as well as deeper states, including V_OH_ and V_O_ in PO_4_^3–^, can participate in transitions that lead to emissions in both higher-energy and lower-energy regions.^24^ Recent experimental data by Raj et al.^7^ further demonstrated that V_O_ within PO_4_^3–^ lead to emissions with maxima at 460 and 630 nm (λ_exc_ = 425 nm). Combined with the higher density of V_Ca_, V_OH_, and V_O_ in PO_4_^3–^ generated during synthesis with increased CO_3_^2–^ content, thermal treatment is also expected to promote the formation of an additional and growing population of V_O_ within PO_4_^3–^ in carbonated HA.^72^ It is also plausible that additional defects arise from partial decarboxylation at 400 and 450 °C or from the reorganization of CO_3_^2–^ between tetrahedral and hexagonal sites, phenomena that are more pronounced in samples with higher CO_3_^2–^ content. These factors may act synergistically as CO_3_^2–^ concentration increases and B-type substitution becomes prevalent, enhancing the emission intensity and activating radiative recombination at lower energies after heat treatment, especially for samples HAS5–450 to HAS8–450.

Our characterizations reveal that another main change occurring between 400 and 450 °C is the elimination of structural water. Water molecules located within the hexagonal channels or associated with V_O_ in PO_4_^3–^ and V_Ca_ interact with the surrounding lattice through hydrogen-bonding or dipolar interactions.^73^ Their removal is expected to exert a more pronounced influence on the photoluminescence properties as their concentration, together with that of vacancy defects (V_Ca_, V_OH_, and V_O_ in PO_4_^3–^), increases with higher levels of CO_3_^2–^. The irreversible loss of these water molecules, along with NH_4_^+^, can alter the bonding character and the electronic density distribution of the remaining species, introducing additional density of defect-related states within the bandgap.^41^ This effect potentially increases the emission intensity after heat treatment while also promoting lower-energy transitions in the red region. Furthermore, it has been show that water act as a quenching agent for defect-related photoluminescence, and its removal reduces nonradiative decay channels, resulting in a net enhancement of emission intensity.^74–76^ As supported by our results, increasing CO_3_^2–^ incorporation also elevates the density of defect sites susceptible to water-induced quenching, such as ionic vacancies and regions of structural disorder in bulk and surface. In this context, the irreversible removal of structural water becomes even more critical, as it minimizes quenching at these optically active sites, thereby amplifying their contribution to the overall photoluminescent response, consistent with our observations for heat-treated carbonated ACP.^48,70^

Together, these mechanisms suggest that the photoluminescence response of carbonated HA after heat treatment is governed by a complex interplay involving bulk and surface defect chemistry, structural water elimination, and carbonate dynamics. These processes jointly modulate the density and energetic distribution of emissive states, ultimately defining the intensity and spectral characteristics of the emission.

### 3.5 Citrate Functionalization and *In Vitro* Assays

HAS8–450 NPs were selected for the *in vitro* assays due to their optimized photoluminescence and highest emission intensity under 488 nm excitation (Figure S6), which corresponds to the excitation wavelength used in the *in vitro* experiments. To enhance their dispersion and colloidal stability, the NPs were functionalized with citrate ions (labeled as HAS8–450-Cit NPs). The TEM images and DLS size distribution profiles (Figure S7a,b) indicated that HAS8–450-Cit NPs had improved dispersion compared to HAS8–450 NPs, confirming effective stabilization upon surface functionalization. The unmodified HAS8–450 NPs displayed a large average hydrodynamic diameter of 472 ± 44 nm and a high polydispersity index (PDI) of 0.576 ± 0.032, which was attributed to their near-neutral surface charge, as indicated by a zeta potential of 4.6 ± 0.2 mV. Following citrate functionalization, the HAS8–450-Cit NPs showed a reduced average hydrodynamic size of 53 ± 5 nm and a lower PDI of 0.292 ± 0.036. This enhancement in colloidal stability was associated with the introduction of negatively charged citrate groups on the NP surface, which resulted in a more negative zeta potential of −17.3 ± 1.0 mV. The photoluminescence emission profile remained similar after functionalization, with only a slight variation in intensity, as shown in Figure S7c for the spectra recorded in aqueous dispersion.

For the *in vitro* assays, HDFn cells were employed as a representative model of healthy human fibroblasts, which are key components of normal connective tissues and are commonly used to investigate the cytocompatibility of HA-based nanomaterials for diverse biomedical applications.^12,77–80^ Moreover, these cells also served to evaluate the cellular internalization of the NPs and their bioimaging capability. Figure 7a presents the cytotoxicity results of HAS8–450-Cit NPs assessed by the MTT assay. No statistically significant differences were observed compared to the control group (cells not exposed to NPs) after 24 h of incubation, confirming the high biocompatibility of the NPs. The cellular uptake of the HAS8–450-Cit NPs was assessed by flow cytometry based on their defect-related photoluminescence emission under 488 nm excitation, following incubation of HDFn cells with 320 μg mL^–1^ of NPs for 4 and 24 h. The geometric mean fluorescence intensity (MFI) is shown in Figure 7b, with statistically significant differences observed between the autofluorescence of the control groups (untreated cells at 4 and 24 h) and the treated groups (****, *p* < 0.0001). These results indicate that the NPs were already internalized by the cells after 4 h and confirm that the photoluminescence of carbonated HA NPs can serve as a tool for monitoring their internalization in cells. In addition, the small but statistically significant difference (****, *p* < 0.0001) observed between 4 and 24 h may be associated with a saturation effect in the internalization process.

**Figure 7:**
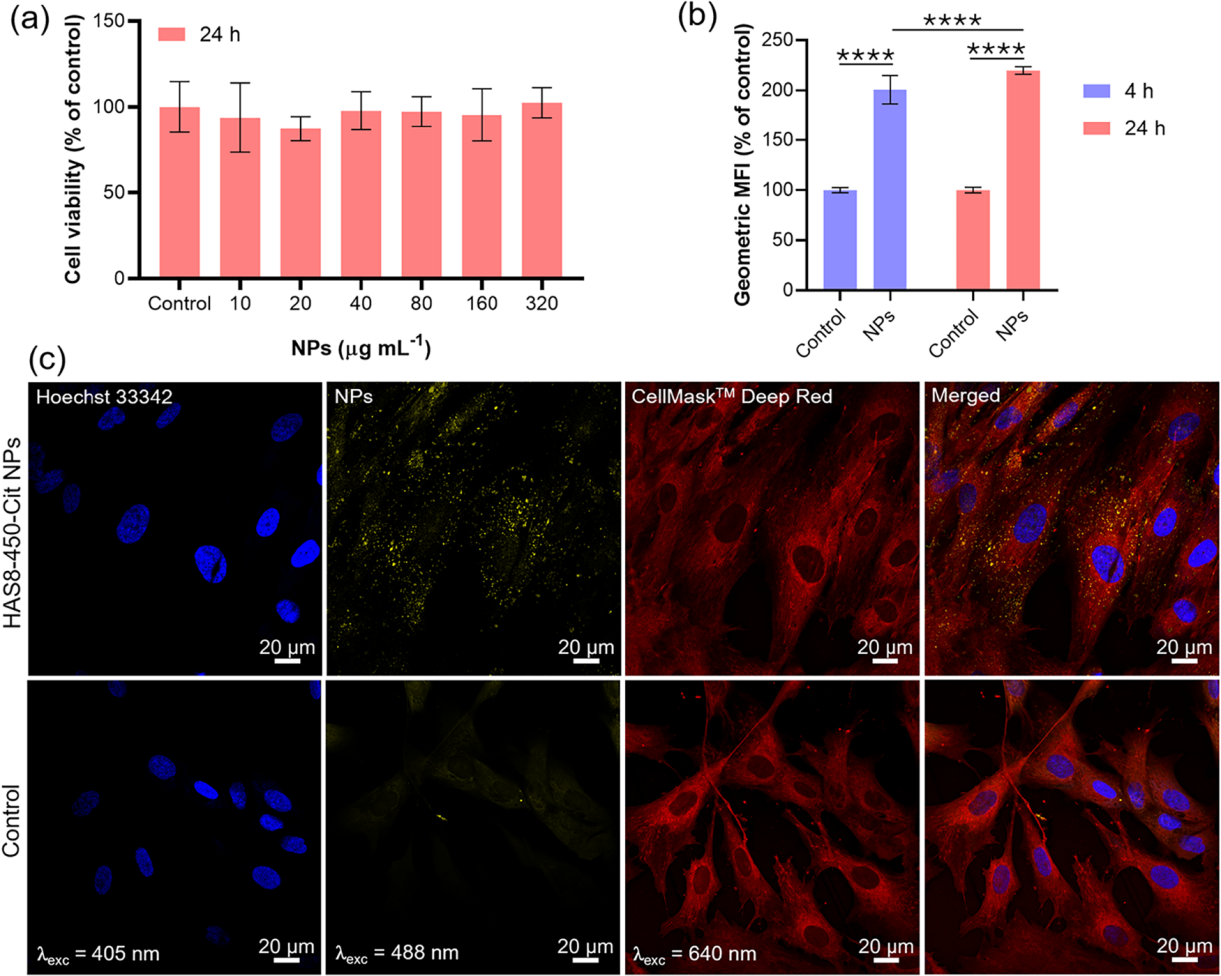
(a) Cell viability of HDFn cells assessed by MTT assay after 24 h of incubation with varying concentrations of citrate-functionalized HAS8–450 NPs, (b) nanoparticle uptake evaluated by flow cytometry after incubation at 320 μg mL^–1^ for 4 and 24 h, and (c) CSLM images of HDFn cells coincubated with citrate-functionalized NPs at 320 μg mL^–1^ for 4 h, acquired using a 40× oil immersion objective. Untreated cells were used as control groups in all experiments. In the flow cytometry analysis, the control geometric MFI corresponds to the autofluorescence of HDFn cells. Statistical significance was determined by one-way (MTT) or two-way (flow cytometry) ANOVA followed by Tukey’s *post hoc* test: ****, *p* < 0.0001.

Figure 7c shows CLSM images of HDFn cells coincubated with HAS8–450-Cit NPs for 4 h at a concentration of 320 μg mL^–1^, captured at an intracellular focal plane. A complete Z-stack analysis is provided in Figure S8. The emission from the NPs, excited at 488 nm and displayed in yellow, is clearly visible and colocalizes with the cytoplasmic region in the merged image, which also shows nucleus in blue (Hoechst 33342) and cell membranes in red (CellMask Deep Red). In contrast, the control cells exhibited only minor autofluorescence under the same excitation conditions, confirming the minimized background signal. These results demonstrate that the intrinsic photoluminescence of the internalized NPs enables intracellular imaging without the need for additional fluorescent labeling.

## 4 Conclusion

New insights into the intrinsic photoluminescence of HA NPs were obtained through a systematic investigation of their structural, compositional, and optical properties. This study establishes CO_3_^2–^ substitution as a key determinant of defect-related photoluminescence in HA NPs. By varying CO_3_^2–^ incorporation from 0.6 to 10.9 wt %, we demonstrated that increased CO_3_^2–^ levels favor B-type substitution over A-type substitution, leading to crystal growth inhibition along with higher structural and surface disorder. These effects enhance the density of radiative defect states, leading to stronger emission in the 438–540 nm range. Thermal treatments at 400–450 °C further amplified the luminescence, with up to a 5-fold increase in intensity and the emergence of red-shifted low-energy emission components, attributed to defect rearrangements, structural water elimination, and reduced nonradiative quenching.

Overall, these results highlight the critical role of carbonate in modulating defect-related photoluminescence in chemically precipitated HA, both before and after thermal processing. Beyond advancing the fundamental understanding of defect structures, these findings support the design of HA-based optical and photocatalytic materials, as well as the defect-sensitive spectroscopic characterization of bone tissue. As a proof of concept, we further demonstrated that the citrate functionalized HA NPs containing 7.8 wt % of CO_3_^2–^ exhibit no significant cytotoxicity, as assessed by the MTT assay in HDFn cells, and can be successfully applied in bioimaging and flow cytometry analysis.

## Supplementary Material



## Notes

Representative TEM images of samples HAS1 to HAS8, as well as HAS8–400 and HAS8–450; Raman spectra of the as-synthesized samples; deconvoluted band assigned to the ν_2_ mode of CO_3_^2–^ observed by FTIR; emission spectra of the as-synthesized samples with an expanded *y*-axis (λ_exc_ = 405 nm); emission spectra of the thermally treated samples at λ_exc_ = 488, 561, and 640 nm; morphological analysis by TEM, size distribution by DLS and emission spectra of the HAS8–450 sample before and after citrate functionalization; Z-stack analysis by CLSM (PDF)
