# Supplementary material for: Defect-Related Photoluminescence in Hydroxyapatite Nanoparticles Modulated by Carbonate Incorporation
Source: ACS Nanosci Au. 2025 Dec 18;6(2):235–49. doi: 10.1021/acsnanoscienceau.5c00140 (PMC13087933; doi:10.1021/acsnanoscienceau.5c00140)
Supplement: Supplementary file 1 [file ng5c00140_si_001.pdf]

SUPPLEMENTARY INFORMATION

Defect-related photoluminescence in hydroxyapatite nanoparticles modulated by carbonate incorporation

Thales R. Machado,[†,*] *Lívia G. Pacífico,*[†] *Marylyn S. Arai,*[†] *Beatriz G.R. da Silva,*[†] *Angélica M.M. Zapata,*[†] *Raquel R.C. Vilela,*[†] *Valtencir Zucolotto*[†,*]

[†]GNANO – Nanomedicine and Nanotoxicology Group, São Carlos Institute of Physics, University of São Paulo, IFSC – USP, 13566-590, São Carlos, SP, Brazil.

*Corresponding authors: Thales R. Machado (trmachado@ifsc.usp.br)
Valtencir Zucolotto (zuco@ifsc.usp.br)

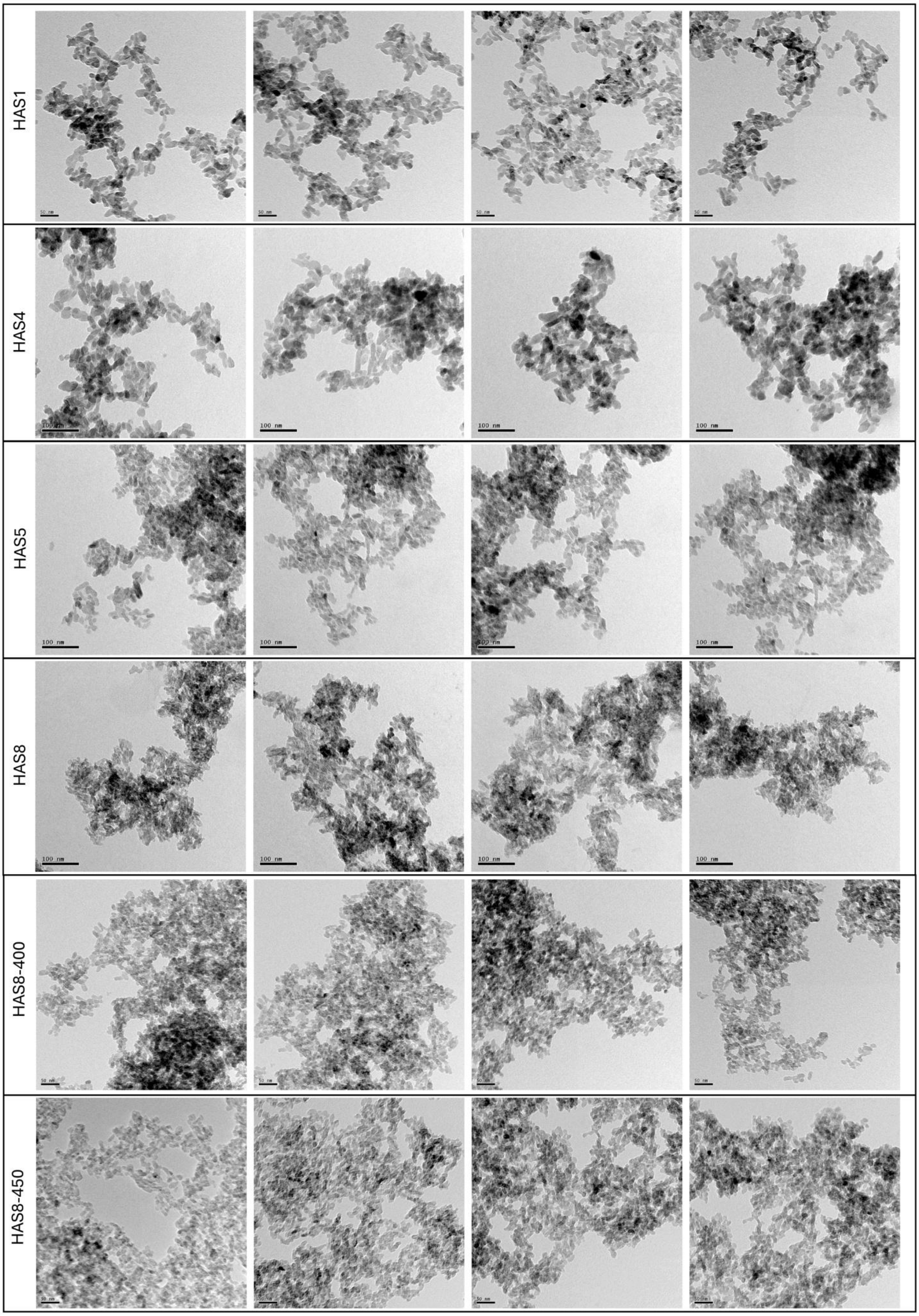

Figure S1. Additional representative TEM images used for the morphological analysis of the as-synthesized carbonated HA NPs.

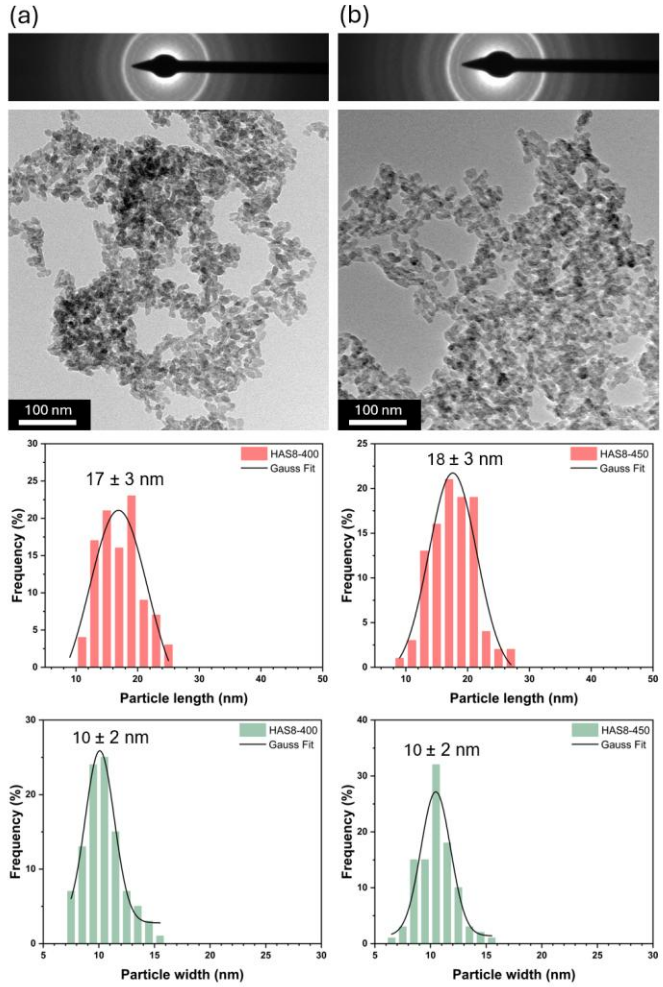

Figure S2. TEM analysis of the heat-treated NPs: (a) HAS8-400, and (b) HAS8-450.

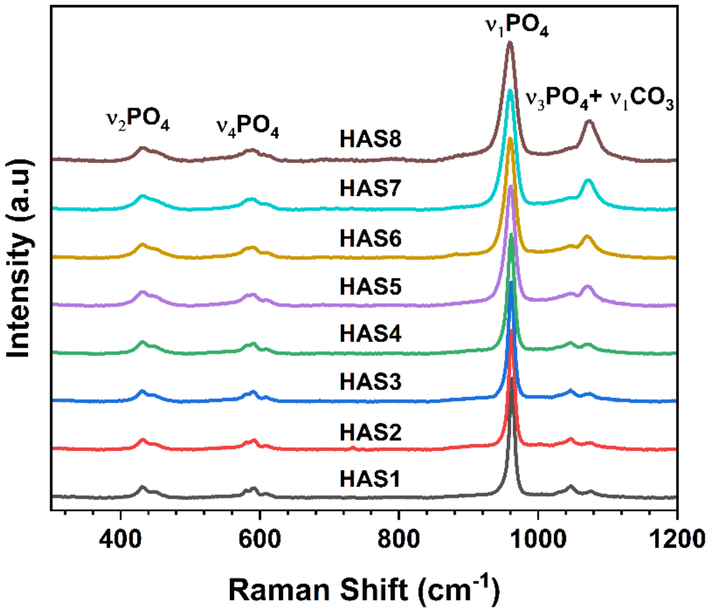

Figure S3. Raman spectra recorded for all as-synthesized HA NPs. All the ν_1, ν_2, ν_3, and ν_4 vibrational modes associated with phosphate groups in the HA structure were observed. In particular, the ν_1 mode at 959 cm^{-1} is characteristic of HA and helps distinguish it from other calcium phosphate phases. Carbonate incorporation was also confirmed by the presence of the ν_1 vibrational band of CO_3^{2-} groups in these spectra, whose intensity increases with carbonate content.

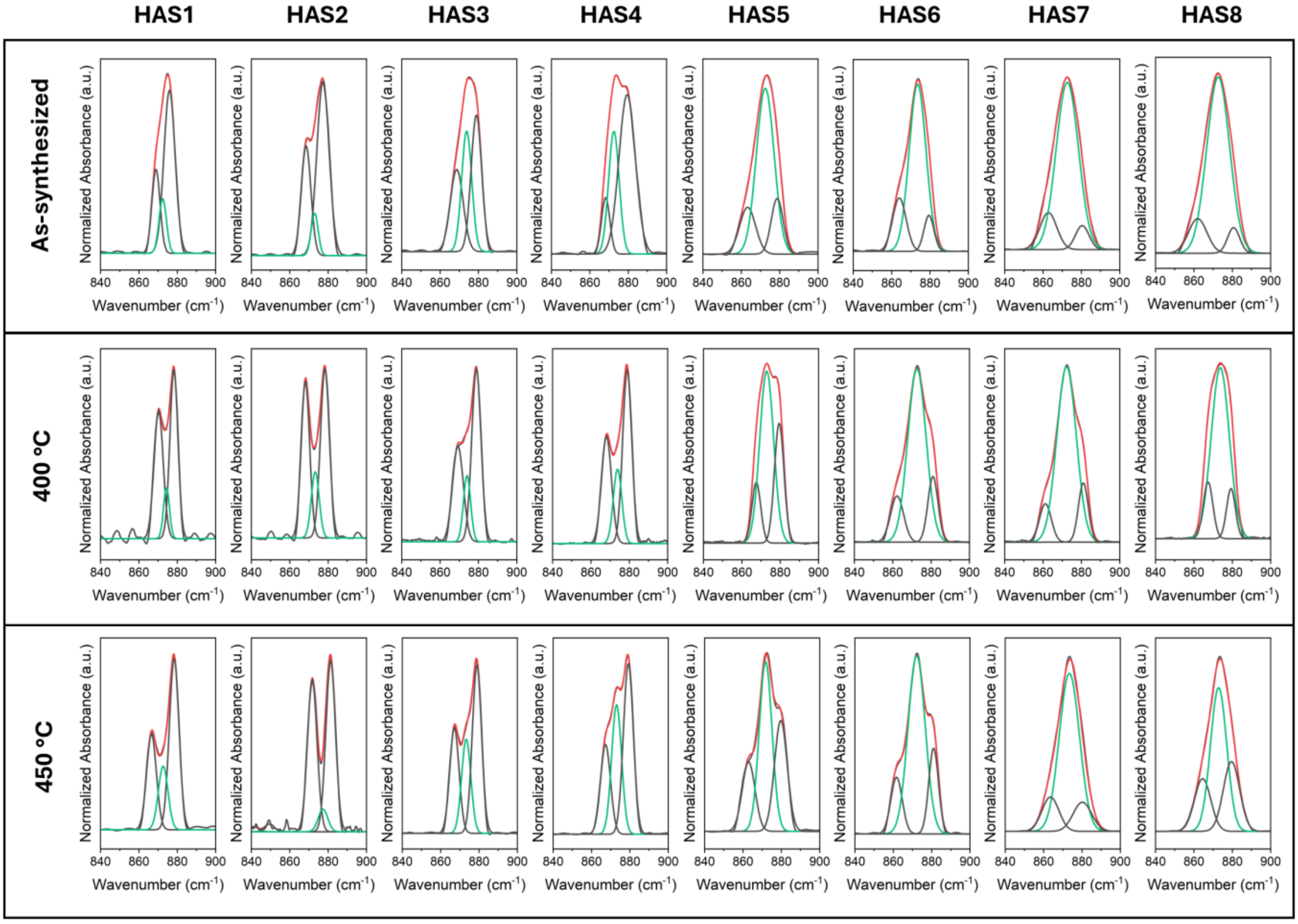

Figure S4. Analysis of the ν_2CO_3 vibrational mode observed by FTIR. The bands were deconvoluted in Origin® (version 2022b) using a Gaussian function after baseline correction. In all cases, the fitting procedure converged well with three components at approximately 864, 873, and 879 cm^{-1}. The component at 873 cm^{-1} (green line) is attributed to B-type CO_3^{2-} substitution, whereas the components at 864 and 879 cm^{-1} (black lines) are associated with A-type CO_3^{2-} in distinct orientations and chemical environments within the hexagonal channels.[1,2] This analysis confirms that all samples correspond to AB-type substitution.

Table S1. Estimated $w(CO_3)$ values via FTIR for carbonated HA samples.

**Sample**	**$[CO_3^{2-}]/[PO_4^{3-}]$ molar ratio**	**$w(CO_3)$ (wt.%) As-Synthesized**	**$w(CO_3)$ (wt.%) 400 ºC**	**$w(CO_3)$ (wt.%) 450ºC**
**HAS1**	-	0.5	0.5	0.4
**HAS2**	0.0625	0.7	0.6	0.8
**HAS3**	0.125	1.3	1.2	1.1
**HAS4**	0.25	1.9	1.7	1.5
**HAS5**	0.5	4.8	3.3	2.9
**HAS6**	1	5.4	3.7	3.2
**HAS7**	2	7.8	6.0	5.5
**HAS8**	4	10.9	8.7	7.9

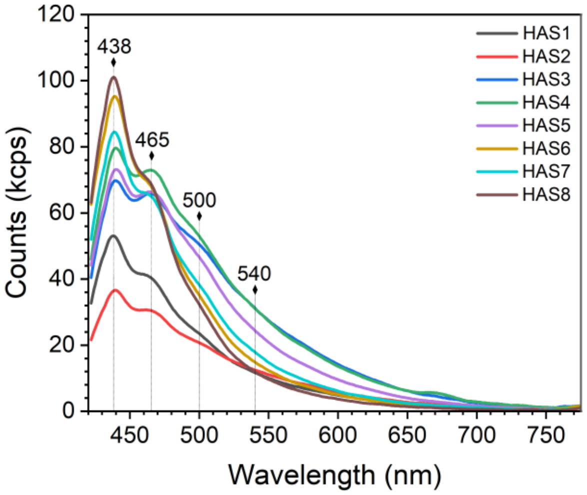

Figure S5. Photoluminescence emission spectra of the as-synthesized carbonated HA NPs (λ_{exc} = 405 nm) shown with a magnified *y*-axis scale.

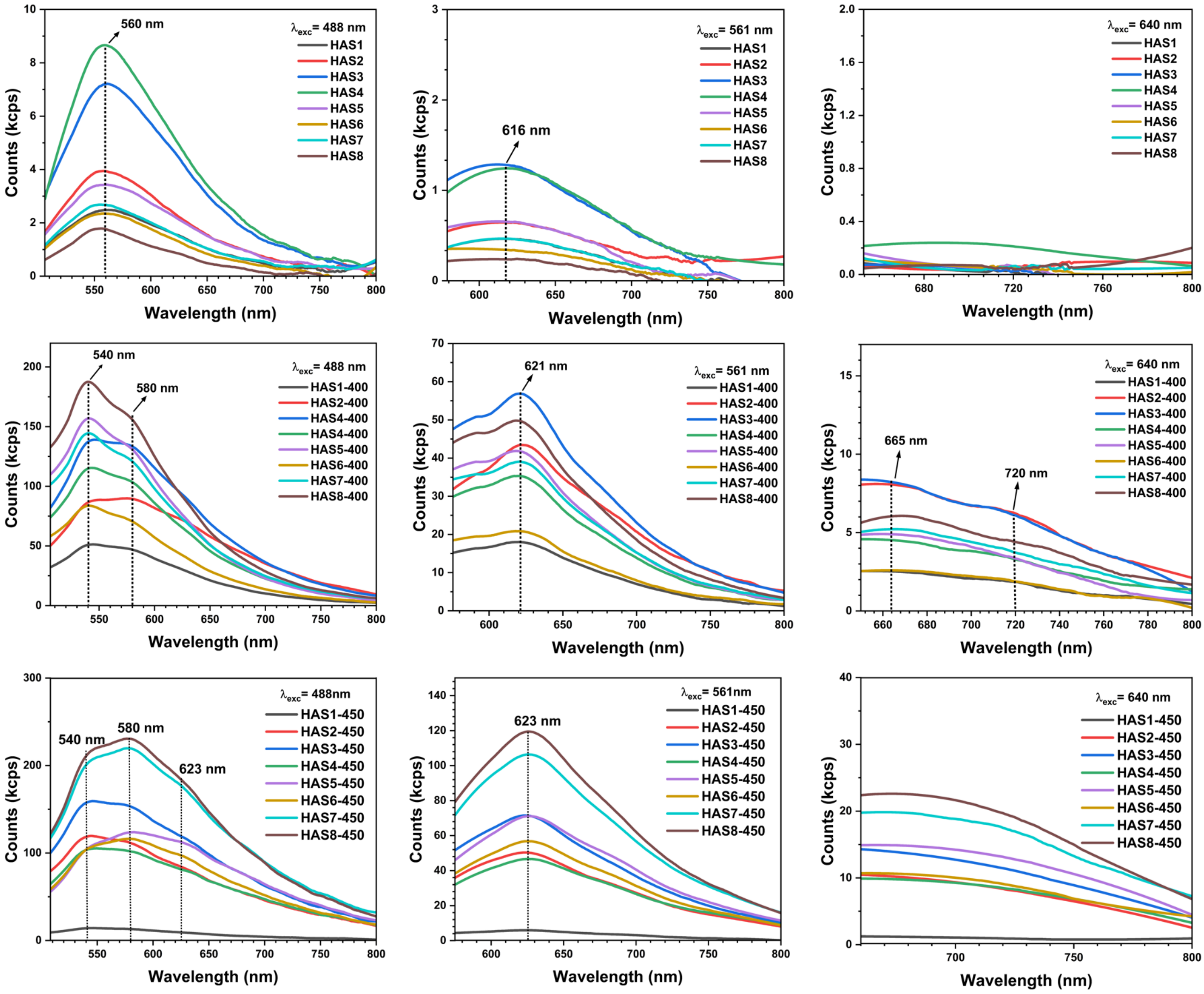

Figure S6. Photoluminescence emission spectra of the samples before and after thermal treatments under excitation at 488, 561, and 640 nm.

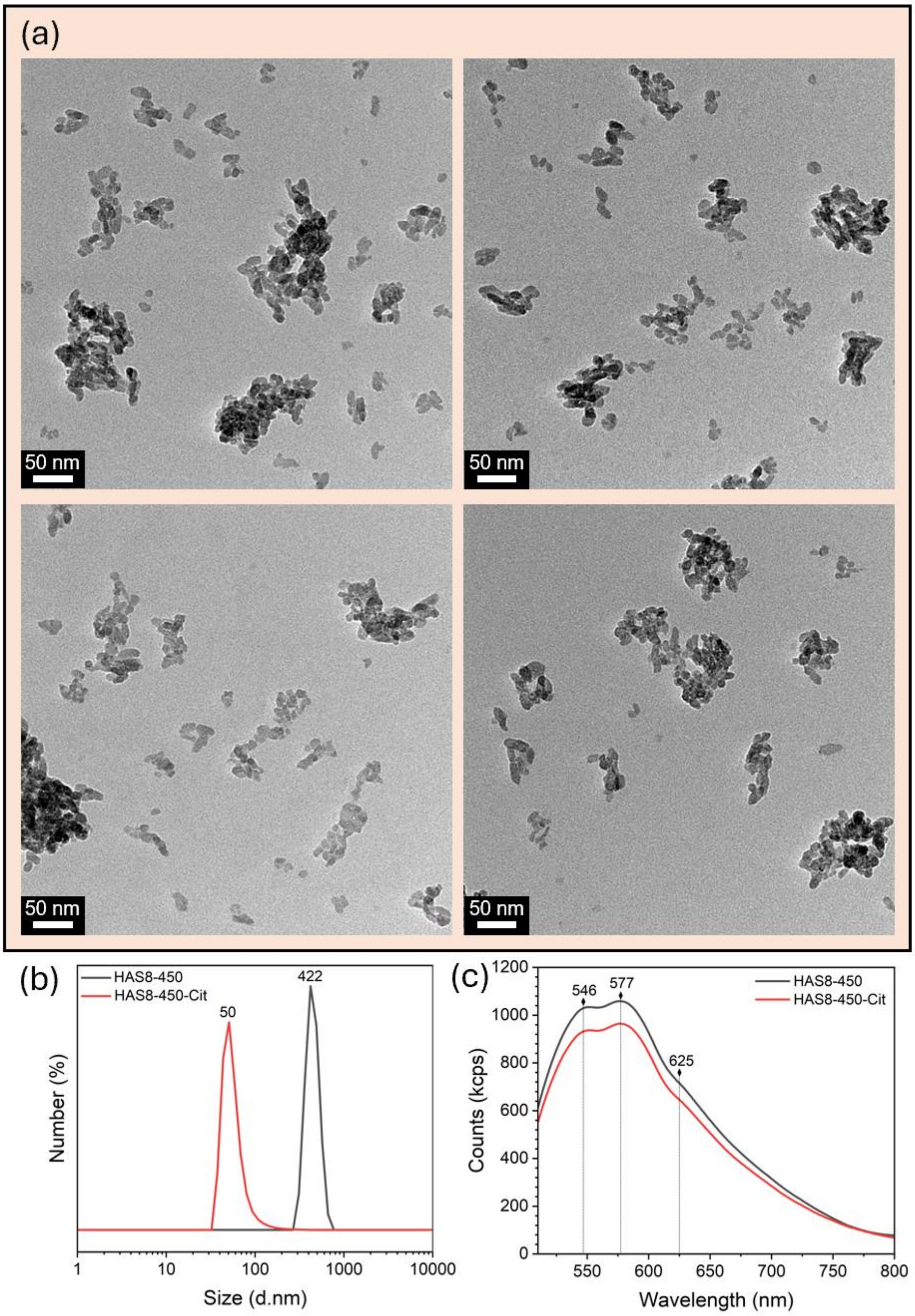

Figure S7. (a) TEM images of HAS8-450-Cit NPs, (b) size distributions of HAS8-450 and HAS8-450-Cit NPs obtained by DLS characterization, and (c) photoluminescence emission spectra of both samples excited at 488 nm in aqueous dispersion at 1 mg mL^{-1}.

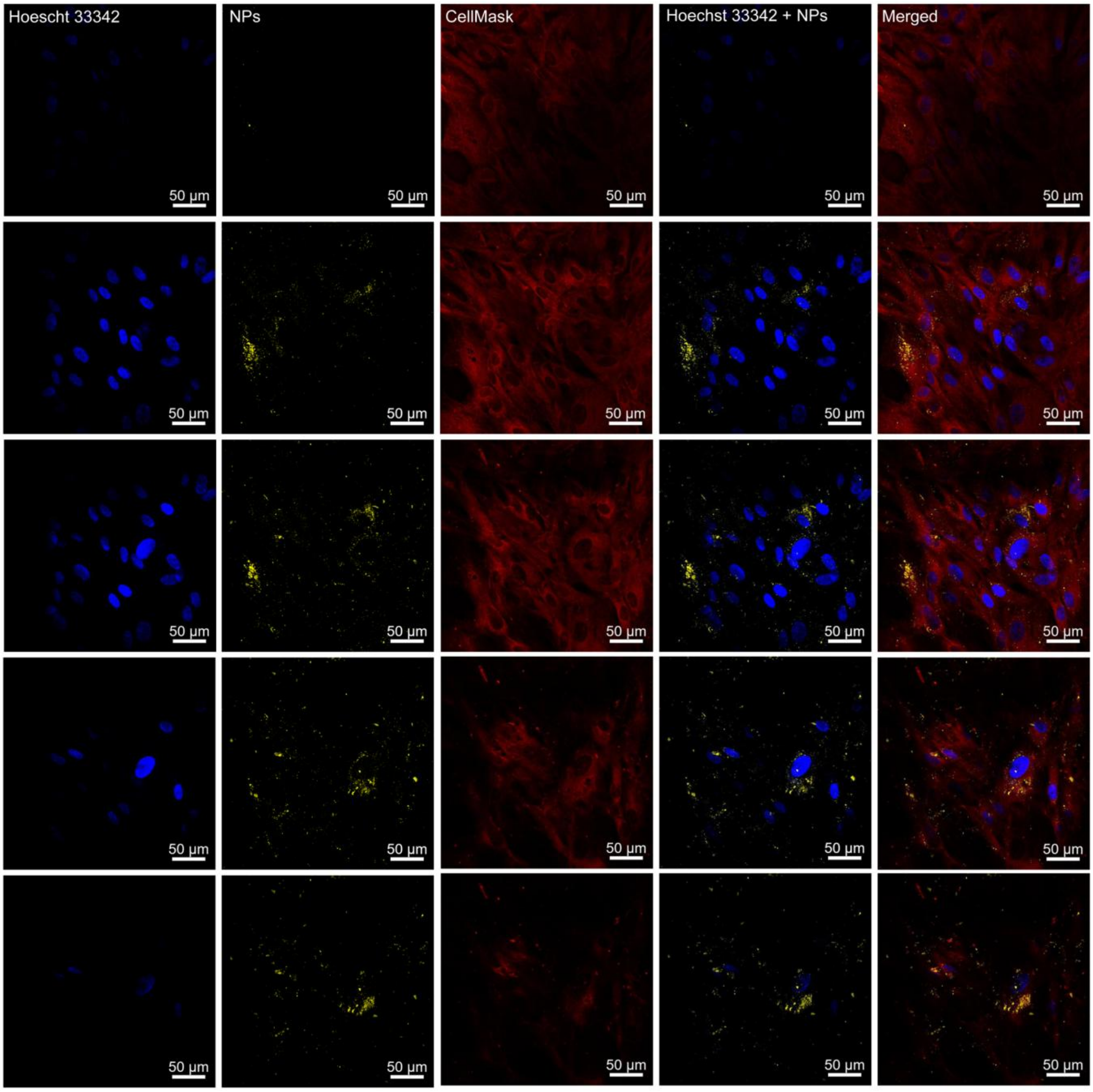

Figure S8. Z-stack analysis by CSLM of HDFn cells co-incubated with citrate-functionalized HAS8-450 NPs at 320 µg mL^{-1} for 4 h. Blue: nuclei (λ_{exc} = 405 nm, Hoechst 33342); yellow: NPs (λ_{exc} = 488 nm); red: cell membrane (λ_{exc} = 640 nm, CellMask^{TM} Deep Red). The focal plane corresponding to the lower cell boundary shows no presence of NPs (stack 1, above). The following optical sections (stacks 2 to 4), corresponding to the cell interior, clearly reveal NPs located adjacent to the nuclear region but not colocalizing with it. These NPs are observed exclusively within these focal planes, thereby confirming their internalization within the cytoplasmic region. In the uppermost focal plane (stack 5, below), NPs are visible at the cell surface, corresponding to a fraction of non-internalized particles.

REFERENCES

(1) Fleet, M. E. Infrared Spectra of Carbonate Apatites: ν_2-Region Bands. *Biomaterials* **2009**, *30* (8), 1473–1481. https://doi.org/10.1016/j.biomaterials.2008.12.007.

(2) Fleet, M. E. *Carbonated Hydroxyapatite : Materials, Synthesis, and Applications*; CRC Press: Boca Raton, 2015.
